# Mass Incarceration, Maternal Vulnerability, and Birth Outcomes Across U.S. Counties

**DOI:** 10.1007/s10995-024-03960-0

**Published:** 2024-07-16

**Authors:** Melanie McKenna, Kathryn M. Nowotny

**Affiliations:** Department of Sociology, University of Miami, Coral Gables, FL USA

**Keywords:** Birth outcomes, Incarceration, Structural racism, Maternal health, Maternal vulnerability

## Abstract

**Objectives:**

To examine the associations among mass incarceration, maternal vulnerability, and disparities in birth outcomes across U.S. counties, utilizing an ecological model and reproductive justice perspective was used. This study tests whether mass incarceration is associated with infant mortality and low birthweight across U.S. counties, and whether maternal vulnerability explains the relationship between mass incarceration and birth disparities.

**Methods:**

Data were derived from a variety of public sources and were merged using federal FIPS codes. Outcomes from the CDC Vitality Statistics include percent low birth weight births (births below 2499 g divided by singleton births to women aged 20 to 39) and infant mortality (infant deaths per 1000 live births). Black–White rate ratios were calculated for the birth outcomes to specifically examine the large Black–White disparity in birth outcomes. The analysis controlled for urbanicity, income inequality, median household income, residential segregation, and southern region, as well as a fixed effect for state level differences.

**Results:**

Findings show that counties with higher rates of incarceration have higher prevalence of infant mortality and low birthweight, as well as greater Black–White disparity in infant mortality. Mass incarceration is associated with increases in adverse birth outcomes and maternal vulnerability partially mediates this relationship.

**Conclusions:**

Findings provide evidence that heightened levels of incarceration affect birth outcomes for all residents at the county-level. It is imperative to address the overuse of mass incarceration in order to support adequate reproductive healthcare of vulnerable populations in the United States.

## Significance

Previous research has found a negative realtionship exists between incarceration and population health, but the extent to which birth outcomes vary by incarceration rate at the county level are unknown.

This study estimates associations between county-level rates of total incarceration, jail admission, and prison admission, on rates of infant mortality and low-birthweight. Results show that prevalence of low-birthweight and infant mortality are increased in U.S. counties with heightened incarceration rates and that this realtionship is partially mediated by maternal vulnerability, an estimation of risk for adverse pregnancy outcomes for women in the area. This stidy also found that mass incarceration contributes to the widening of Black-White disparities in infant mortality.

## Introduction

Racism has been well established as a structural determinant of health that is both fundamental and pervasive in nature, barring equal distribution of resources including access to healthcare for marginalized populations and people living in disinvested areas. Black women, specifically, face the “double jeopardy” of both racial and gender oppression, which uniquely impacts their health (Geronimus et al., 2006). In the United States, there is a persistent history of reproductive oppression which has long devalued the Black female body. Reproductive oppression remains integrated into modern structural and institutional practices that maintain racial inequality in maternal and infant health today (Taylor, 2020; Washington, 2006). Structural racism is evidenced in birth and infant outcomes. The rate of singleton low birthweight was more than twice as high for non-Hispanic Black infants than for non-Hispanic White infants from 2006 to 2016 (Womack et al., 2018). In 2018, the rate per 1000 live births of infants that died before their first year of life was 10.8 for African Americans and 4.9 for Whites (non-Hispanic unless otherwise noted), (CDC, 2018).

Scholars have identified four mechanisms through which structural racism impacts health: (1) civil rights laws and legal racial discrimination; (2) residential segregation and housing discrimination; (3) police violence; and (4) mass incarceration (Alson et al., 2021). Of these, mass incarceration has arguably been the lesser studied indicator of structural inequity in relation to reproductive health (LeMasters et al., 2022). Previously, structural racism has been measured as racial inequity in educational attainment, median household income, employment, imprisonment, and juvenile custody, and has been found to be associated with higher infant mortality rates for infants born to Black women (Wallace et al., 2017).

However, there is overwhelming evidence that mass incarceration negatively impacts the health of justice involved people, their loved ones, and their communities. Some of the ways in which effects of incarceration “spillover” to communities to create adverse outcomes at the population level, are through racist discriminatory policies that prevent access to housing, employment, and health care (Bailey et al., 2017). States with the largest incarcerated populations also have the lowest access to care and lowest quality of care, partly due to previously incarcerated people being denied health coverage, leaving them without adequate access to healthcare (Schnittker et al., 2015). High incarceration rates in U.S. counties and communities negatively affect the health of all residents. Incarceration reduces social support by removing family and community members, as well as increases the presence of incarceration-related stigma, disenfranchisement, and higher levels chronic stress (Alson et al., 2021). Concentrated incarceration also causes increased economic strains through lost earnings coupled with the burden of costs to financially support an incarcerated loved one (Braman, 2004). Stigma and resulting social isolation caused by having a family member incarcerated is an important social stressor indirectly impacting health through negative coping behaviors (Braman, 2004).

Previous studies have linked mechanisms of structural racism to health at the population level to document the effect of incarceration on reproductive healthcare outcomes. For instance, U.S. county-level differences in incarceration rates are associated with increased sexually transmitted infection (STI) incidence (Dauria et al., 2015; Nowotny et al., 2020; Thomas & Sampson, 2005) and risk of preterm birth (Jahn et al., 2020). U.S. counties with higher incarceration rates have increased rates of teenage pregnancies (Thomas & Torrone, 2008) and, at the state level, the imprisonment rate is positively associated with infant mortality rates, Black–White inequality in infant mortality rates, and Black–White inequality in life expectancy at birth (Wildeman, 2009). Examining community-level characteristics is necessary to understand the relationship between incarceration as a mechanism of structural racism, and adverse reproductive health outcomes for women.

Prisons and jails are very different spaces and have different population trends. Prison populations tend to be more stable because people held in prisons have generally been convicted of a felony offense and are serving a sentence. On any given day there are about 1.5 million people in prison operated by states and the federal government. Jails, on the other hand, have a higher population turnover. On any given day there are about 600,000 people in U.S. county-operated jails, yet 10 million people are admitted to jails each year. Most people held in county jails have not been convicted of a crime and are waiting for arraignment or the adjudication of their case. The average length of stay in jail is 26 days (BJS, 2017) while average stay in prison is 2.7 years (BJS, 2018). The more transient population of those admitted and discharged from jails leads to less time for medical evaluation and care, as well as frequent reentry to the community. However, those admitted to prison serve longer sentences and are missing from their families and communities for greater amounts of time. Unlike previous studies, we examine whether jail and prison county-level rates differentially impact birth outcomes.

We hypothesize that mass incarceration negatively affects birth outcomes as well as overall maternal vulnerability. Maternal vulnerability is a sociostructural indicator that varies across U.S. counties and is associated with reproductive healthcare, physical health, mental health and substance abuse, general healthcare, socioeconomic determinants, and the physical environment (Surgo Ventures, 2021). We utilize the Maternal Vulnerability Index county-level scores (Surgo Ventures, 2021), which identifies where and why birthing people are vulnerable to poor health outcomes. The Maternal Vulnerability Index scores have been used to predict county-level risk for adverse birth outcomes, such as preterm birth, and has been shown to have policy implications for improving perinatal outcomes (Salazar et al., 2023). Therefore, this study aims to: (1) test whether county-level incarceration rates and maternal vulnerability are associated with higher rates of overall negative birth outcomes, including higher percentage of births that are of low birthweight and higher rate of infant mortality; (2) examine the association between incarceration rates and maternal vulnerability on Black–White disparities in negative birth outcomes; and (3) test the mediating effect of maternal vulnerability on mass incarceration and negative birth outcomes.

## Methods

We used data from multiple existing sources to examine the relationship among mass incarceration, maternal vulnerability, and Black–White disparities in birth outcomes across U.S. counties. The county-level dataset was created using Federal Information Processing Standards (“FIPS codes”). The two birth outcomes included in the study are infant mortality rate and low birth weight. Infant mortality rate represented the number of infant deaths for every 1000 live births and was calculated using the Linked Birth/Death Records 2017–2018 from the Centers for Disease Control and Prevention (National Center for Health Statistics, National Vital Statistics System) for a total infant mortality rate per county, and a mortality rate for infants born to Black women and White women, respectively, per county. Low birthweight was defined as a birthweight less than 5.5 pounds (2499 g). Data for the most recent time period available was used. We used pooled CDC Vitality Statistics Public Access Files for data on births from 2007 to 2019, for infants born in U.S. counties with populations over 100,000 people, We restricted the low birthweight outcome further to include only singleton first births born to women 20–39 years of age. low birthweight was calculated as the percentage of births in the county within a calendar year that resulted in a low birthweight newborn over the total number of births for Black and White women. Black–White rate ratios were calculated for each outcome to directly assess Black–White disparities. A ratio above 1.0 means that the rate for Black women was higher than for White women, below 1.0 means that the rate for Black women was lower than for White women.

Incarceration rates per county were obtained from the Vera Institute of Justice and included total incarceration, prison incarceration, and jail incarceration per 100,000 people using average daily population estimates at the U.S. county-level throughout 2020 and Spring 2021 (Kang-Brown et al., 2021). Maternal vulnerability was measured using the Maternal Vulnerability Index (MVI; Surgo Ventures, 2021), a score per county on a scale of 0–100 (0 = least vulnerability, 100 = most vulnerability). Total MVI is comprised of 6 categories, each of which are defined further by subcategories: reproductive healthcare (4 items), physical health (6 items), mental health and Substance Abuse (4 items), general healthcare (4 items), socioeconomic determinants (5 items), and physical environment (4 items). See supplementary material for more details. Data for each subcategory was obtained from governmental and other sources such as the American Community Survey (ACS), National Survey on Drug Use and Health (NSDUH), Centers for Disease Control and Prevention (CDC), Guttmacher Institute, Kaiser Family Foundation, and state and county health departments.

Covariates included urbanicity, income inequality, median household income, racial residential segregation, and region. Urbanicity codes included rural, small, mid, suburban, or urban by the Department of Agriculture (Ratcliffe et al., 2016; U.S. Census Bureau, 2020). Rates of income inequality, median household income, and racial residential segregation were obtained from The Robert Wood Johnson Foundation County Health Rankings and Roadmaps database, which consolidates data from governmental sources (e.g., American Community Survey, United States Department of Agriculture, Centers for Disease Control and Prevention). Income inequality was the ratio of household income at the 80th percentile to income at the 20th percentile (data from 2015 to 2019). The U.S. counties for which a full study dataset was available were in 29 states, with all four Census regions represented: 20% of U.S. counties were located in the Midwest, 18% in the Northeast, 52% in the South, and 10% in the West. We included a dummy variable for the southern region.

The number of U.S. counties included in our study was restricted based on the birth outcomes. First, only U.S. counties with populations over 100,000 were included. Second, our sample was restricted to the 350 U.S. counties for which we had data on infant mortality outcomes of Black women, then this sample size was further reduced as 83 additional U.S. counties were dropped due to missing incarceration data and a few more U.S. counties were dropped due to missing data for the control variables, leaving 266 U.S. counties. For the low birthweight outcomes, 335 U.S. counties had data for low birthweight percentages of infants to Black women. Incarceration data was missing for 91 U.S. counties and a few more U.S. counties were dropped due to missing data for the control variables. Thus, the final sample size was 266 U.S. counties for infant mortality outcomes and 244 U.S. counties for low birthweight outcomes. To test if there was a significant difference between missing and non-missing U.S. counties with a population over 100,000, a series of t-test were conducted. None of the means were significantly different, therefore, the missing data should not bias results.

**Fig. 1:**
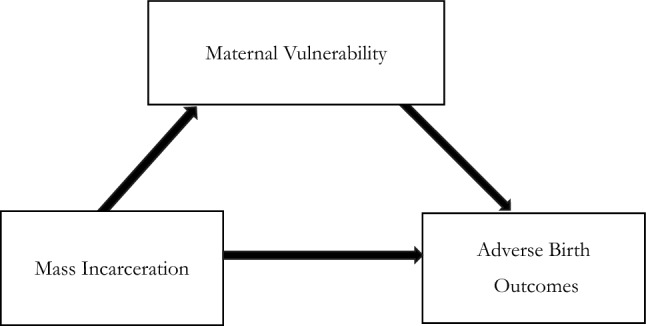
Mediation path diagram

A descriptive analysis included means, standard deviations, frequencies, and ranges. A bivariate analysis was performed using Pearson *r* coefficients. Multiple regression analysis (OLS) examined the association of low birthweight and infant mortality with incarceration and maternal vulnerability, while controlling for income inequality, median household income, residential segregation, urbanicity, and southern region. Models included state-level fixed effects. A test of multicollinearity produced a VIF of 1.58 with no individual score over 2. Standardized coefficients, the *β* values, were reported. We estimated the association of total incarceration, controlling for covariates, on total infant mortality (Model 1), infant mortality Black–White ratio (Model 2), total low birthweight (Model 3), and low birthweight Black–White rate ratio (Model 4). Models 5–8 estimated the association of total incarceration on the same four outcomes with maternal vulnerability added to the model. Models 9–12 estimated the association of total incarceration and the six maternal vulnerability subcategories with infant mortality (Model 9), and its’ Black–White rate ratio (Model 10), as well as low birthweight total (Model 11), and its’ Black–White rate ratio (Model 12). The final models (Models 13–20) estimated the effects of jail and prison admission separately (excluding total incarceration rates) on the birth outcomes and birth outcome ratios. Mediation analysis followed Baron and Kenny (1986) since the predictor, mediator, and outcomes are continuous (Fig. 1). The mediation analysis examined whether the incarceration rate affected the birth outcomes directly, or through a mediator, maternal vulnerability.

## Findings

Table 1 shows the descriptive statistics for the study variables. The average infant mortality rate ratio was 2.57, meaning that singleton infants born to Black women ages 20–39 were over 2 and a half times more likely to die before 1 year of life than their White counterparts. Infants born to Black women were also 1.84 times more likely to be low birthweight (under 2499 g) than their White counterparts. The average total incarceration rate (inclusive of prisons and jails) across U.S. counties was 684 per 100,000. Total incarceration rates per U.S. county had high variation ranging from < 1 to 1877. The range for maternal vulnerability scores were also highly variable at 0.92–97.17, with an average of 49.14.

**Table 1:** Descriptive statistics of main study variables

Variables	No. of obs.	Mean/rate	SD	Min	Max
Incarceration rate	282	683.73	320.93	127.05	1876.83
Prison rate	282	441.09	216.97	57.50	1439.23
Jail rate	282	242.64	136.69	53.14	906.48
Maternal vulnerability	282	49.14	26.02	0.92	97.17
Theme 1: Reproductive health	282	34.68	22.41	0.19	93.47
Theme 2: Physical health	282	54.15	25.53	2.48	97.93
Theme 3: Mental health	282	48.61	24.72	0.25	98.22
Theme 4: General healthcare	282	36.22	27.56	0.10	93.98
Theme 5: Socioeconornic	282	54.29	22.71	4.52	98.69
Theme 6: Physical environment	282	68.37	22.05	5.51	99.87
LBW %	268	7.98	1.48	4.66	14.40
Infant mort. %	282	6.47	1.86	2.75	12.50
LBW BW ratio	244	1.84	0.50	0.78	3.89
Infant mort. BW ratio	266	2.57	0.91	1.08	7.28
Income inequality	282	4.70	0.69	3.51	9.15
Med. house. income	282	60,512.62	15,039.00	33,714.00	118,468.00
Residential seg.	282	48.46	12.36	20.82	79.36
Urbanicity	282	1.63	0.80	0.00	3.00
Southern region	282	0.52	0.50	0.00	1.00

Pearson correlation coefficients, *r,* tested the association between all variables (Table 2). Incarceration rate was positively correlated with maternal vulnerability (*r* = 0.65, p ≤ 0.001), infant mortality (*r* = 0.62, p ≤ 0.001), low birth weight (*r* = 0.46, p ≤ 0.001), and income inequality (*r* = 0.15, p ≤ 0.01), and it was negatively correlated with household income (*r* =  − 0.63, p ≤ 0.001) and urbanicity (*r* = − 0.27, p ≤ 0.001). Maternal vulnerability had a strong and positive correlation with infant mortality (*r* = 0.70, p ≤ 0.001) and percentage of low birthweight (*r* = 0.55, p ≤ 0.001).

**Table 2:** Pearson correlation matrix

Variables	1	2	3	4	5	6	7	8	9	10	11	12	13	14	15	16	17
(1) Incarceration rate	1																
(2) MVI	0.65***	1															
(3) Theme 1: Reproductive health	0.29***	0.53***	1														
(4) Theme 2: Physical health	0.58***	0.88***	0.29***	1													
(5) Theme 3: Mental health	0.44***	0.72***	0.32***	0.67***	1												
(6) Theme 4: General healthcare	0.35***	0.68***	0.54***	0.47***	0.30***	1											
(7) Theme 5: Socioeconornic	0.62***	0.77***	0.01*	0.71***	0.42***	0.35***	1										
(8) Theme 6: Physical environment	0.46***	0.60***	− 0.05	0.55***	0.30***	0.10*	0.69***	1									
(9) % LBW	0.46***	0.55***	0.12*	0.61***	0.40***	0.29***	0.46***	0.42***	1								
(10) Infant mort	0.62***	0.70***	0.13**	0.76***	0.57***	0.30***	0.65***	0.53***	0.60***	1							
(11) Income ineq	0.15**	0.12**	− 0.25***	0.13**	− 0.03	− 0.13*	0.41***	0.42***	0.17***	0.14**	1						
(12) Med. house. income	− 0.63***	− 0.76***	− 0.0.21***	− 0.74***	− 0.61***	− 0.37***	− 0.80***	− 0.52***	− 0.49***	− 0.73***	− 0.12*	1					
(13) Residential seg.	− 0.06	− 0.25***	− 0.33***	− 0.14**	− 0.05	− 0.51***	00.02	00.05	− 0.05	00.04	0.39***	00.04	1				
(14) Urbanicity	− 0.27***	− 0.32***	− 0.21***	− 0.32***	− 0.23***	− 0.20***	− 0.27***	− 0.08	− 0.22***	− 0.32***	0.15**	0.42***	0.24***	1			
(15) Southern region	0.38***	0.64***	0.48***	0.56***	0.39***	0.72***	0.31***	0.15**	0.36***	0.34***	− 0.10*	− 0.38***	− 0.49***	− 0.23***	1		
(16) Prison rate	0.94***	0.64***	0.30***	0.56***	0.41***	0.35***	0.63***	0.44***	0.45***	0.62***	0.15**	− 0.64***	− 0.02	− .23***	0.33***	1	
(17) Jail rate	0.85***	0.52***	0.20***	0.48***	0.37***	0.26***	0.46***	0.39***	0.35***	0.48***	0.12**	− 0.46***	− 0.09	− 0.27***	0.37***	0.63***	1

***p < 0.01; **p < 0.05, *p < 0.10

U.S. county-level total incarceration rates were associated with higher maternal vulnerability scores for all six subcategories, with the strongest correlation coefficients for SES (*r* = 0.62, p ≤ 0.001) and physical health (*r* = 0.58, p ≤ 0.001). The study outcomes were most strongly correlated with the physical health subcategory: (low birthweight (*r* = 0.61, p ≤ 0.001) and infant mortality (*r* = 0.76, p ≤ 0.001)). This subcategory included indicators of prevalence of non-communicable diseases (hypertension, obesity, and diabetes) and sexually transmitted diseases (STIs) (gonorrhea, syphilis, chlamydia, Hepatitis B, and HIV), as well as an indicator of self-rated health.

The multivariate models showed a significant association between total incarceration rate and overall infant mortality (*β* = 0.251, p ≤ 0.001; Model 1), overall low birthweight (*β* = 0.192, p ≤ 0.01; Model 3), and the Black–White ratio for low birthweight (*β* = 0.183, p ≤ 0.05; Model 4); however, the association of total incarceration rate and the infant mortality Black–White ratio was not significant (Model 2). Total incarceration rate (*β* = 0.186, p ≤ 0.001; Model 5) and maternal vulnerability (*β* = 0.354, p ≤ 0.001; Model 5) were both significantly associated with infant mortality. Total incarceration rate and maternal vulnerability were also both significantly associated with low birthweight (*β* = 0.143, p ≤ 0.05 and *β* = 0.29, p ≤ 0.01, respectively; Model 7) (Tables 3, 4).

**Table 3:** Regression models of incarceration rate on infant mortality and low birthweight

	Model 1			Model 2			Model 3			Model 4		
	Infant mortality			Infant mort. BW ratio			LBW			LBW BW ratio		
	Beta	SE	p	Beta	SE	p	Beta	SE	p	Beta	SE	p
*Main effect*												
Incarceration rate	0.251	0	***	− 0.038	0		0.192	0	**	0.183	0	*
*Controls*												
Income inequality	0.026	0.122		0.034	0.083		0.106	0.122		− 0.127	0.05	
Med. house. income	− 0.524	0	***	0.003	0		− 0.293	0	***	0.257	0	**
Res. segregation	0.096	0.008		0.301	0.005	***	0.049	0.008		− 0.033	0.003	
Urbanicity	− 0.049	0.105		− 0.072	0.072		− 0.116	0.106	*	− 0.013	0.044	
State	0.007	0.005		− 0.018	0.003		− 0.026	0.005		0.03	0.002	
Southern region	0.091	0.188		− 0.214	0.128	**	0.177	0.194	**	0.141	0.08	
N	266			266			244			244		
r2	59%			20%			36%			8%

**Table 4:** Regression models of incarceration rate and maternal vulnerability on infant mortality and low birthweight

	Model 5			Model 6			Model 7			Model 8		
	Infant mortality			Infant mort. BW ratio			LBW			LBW BW Ratio		
	Beta	SE	p	Beta	SE	p	Beta	SE	p	Beta	SE	p
*Main effect*												
Incarceration rate	0.186	0	***	0.074	0		0.143	0	*	0.188	0	*
Maternal vulnerability	0.354	0.006	***	− 0.612	0.004	***	0.29	0.006	**	− 0.028	0.003	
*Controls*												
Income inequality	− 0.01	0.12		0.096	0.08		0.085	0.121		− 0.125	0.051	
Med. house. income	− 0.34	0	***	− 0.315	0	***	− 0.138	0		0.242	0	*
Res. segregation	0.131	0.008	**	0.241	0.005	***	0.064	0.008		− 0.034	0.003	
Urbanicity	− 0.061	0.101		− 0.051	0.068		− 0.125	0.105	*	− 0.012	0.044	
State	0.009	0.005		− 0.022	0.003		− 0.028	0.005		0.031	0.002	
Southern region	− 0.032	0.207		− 0.001	0.138		0.072	0.222		0.151	0.093	
N	266			266			244			244		
r2	62%			29%			38%			8%		

Maternal vulnerability subcategories were inconsistently associated with birth outcomes. Reproductive health, the first subcategory, was significantly associated with infant mortality (*β* = − 0.127, p ≤ 0.01; Model 9) and infant mortality Black–White ratio (*β* = − 0.181, p ≤ 0.01; Model 10), indicating that increased reproductive vulnerability was related to decreased infant mortality rate and rate ratio. The second subtheme, physical health vulnerability, was significantly associated with infant mortality (*β* = 0.498, p ≤ 0.001; Model 9), infant mortality Black–White ratio (*β* = 0.247, p ≤ 0.05; Model 10), and low birthweight (*β* = 0.401, p ≤ 0.001; Model 11), and socioeconomic vulnerability was significantly associated with infant mortality Black–White ratio (*β* = − 0.255, p ≤ 0.05; Model 10) and low birthweight (*β* = − 0.286, p ≤ 0.05; Model 11). Lastly, physical environment vulnerability was significantly associated with infant mortality Black–White ratio (*β* = − 0.201, p ≤ 0.01; Model 10), low birthweight (*β* = 0.184, p ≤ 0.05; Model 11), and low birthweight Black–White ratio (*β* = 0.215, p ≤ 0.05; Model 12). Mental health/substance abuse vulnerability and general healthcare vulnerability were not associated with birth outcomes (Tables 5, 6).

**Table 5:** Regression models of incarceration rate and MVI themes on infant mortality and low birthweight

	Model 9			Model 10			Model 11			Model 12		
	Infant mortality			Infant mortality BW ratio			LBW			LBW BW ratio		
	Beta	SE	p	Beta	SE	p	Beta	SE	p	Beta	SE	p
Incarceration rate	0.218	0	***	0.096	0		0.183	0	**	0.159	0	
Th1 Reprod. healthcare	0.127	0.004	**	0.181	0.003	**	0.095	0.004		0.093	0.002	
Th2 Physical health	0.498	0.005	***	0.247	0.004	*	0.401	0.006	***	0.057	0.002	
Th3 Mental health	0.041	0.004		0.08	0.003		0.037	0.004		0.107	0.002	
Th4 General healthcare	0.069	0.004		0.04	0.003		0.112	0.004		0.081	0.002	
Th5 Socioeconomic	0.118	0.007		0.255	0.005	*	0.286	0.008	*	0.105	0.003	
Th6 Phys. environment	0.081	0.004		0.201	0.003	**	0.184	0.005	*	0.215	0.002	*
Income inequality	0.034	0.128		0.175	0.094	*	0.084	0.14		0.174	0.061	*
Med. house. income	0.276	0	***	0.414	0	***	0.18	0		0.172	0	
Res. segregation.	0.086	0.007		0.269	0.005	***	0.05	0.008		0.018	0.004	
Urbanicity	0.054	0.093		0.062	0.068		0.13	0.103	*	0.013	0.045	
Southern region	0.08	0.213		0.085	0.156		0.048	0.238		0.204	0.104	
N	266			266			244			244		
r2	70%			33%			45%			12%

**Table 6:** Regression models of prison and jail rate on infant mortality and low birthweight

	Model 13			Model 14			Model 15			Model 16		
	LBW			Infant mort.			LBW			Infant Mort.		
	Beta	SE	p	Beta	SE	p	Beta	Std. Error	p	Beta	Std. Error	P
Prison rate	0.155	0	*	0.172	0	***						
Jail rate							0.064	0.001		0.11	0.001	*
MVI	0.28	0.006	**	0.39	0.006	***	0.329	0.006	**	0.435	0.006	***
Income ineq.	0.087	0.121		− 0.047	0.121		0.086	0.122		− 0.05	0.122	
Med. house. income	− 0.129	0	*	− 0.31	0	***	− 0.172	0		− 0.351	0	***
Res. segregation	0.064	0.008		0.183	0.008	***	0.069	0.008		0.186	0.008	***
Urbanicity	− 0.132	0.105	*	− 0.084	0.105		− 0.12	0.106	*	− 0.067	0.106	
Southern region	0.083	0.222	*	− 0.06	0.222		0.066	0.225		− 0.084	0.226	
N	244			228			244			228		
r2	38%			62%			38%			61%		

Prison admission rates were significantly associated with low birthweight (*β* = 0.155, p ≤ 0.05; Model 13) and infant mortality (*β* = 0.172, p ≤ 0.001; Model 14), while jail admission rates were significantly associated with infant mortality (*β* = 0.11, p ≤ 0.05; Model 16) and low birthweight Black–White ratio (*β* = 0.163, p ≤ 0.05; Model 19). The R^2^ values for the jail and prison admission rates were much higher for the birth outcome models than for the birth outcome ratio models, indicating using the total birth outcomes was a better fit to describe the variance, and also suggests that separating incarceration rate into jail and prison admission, rather than a total measure, more clearly explains the increase in infant mortality and low birthweight (Tables 6, 7).

**Table 7:** Regression models of prison and jail rate on infant mortality and low birthweight ratios

	Model 17			Model 18			Model 19			Model 20		
	LBW BW ratio			Infant mort. BW ratio			LBW BW ratio			Infant mort. BW ratio		
	Beta	SE	p	Beta	SE	p	Beta	SE	p	Beta	SE	p
Prison rate	0.144	0		0.145	0							
Jail rate							0.163	0	*	0.055	0	
MVI	− 0.016	0.003		− 0.514	0.004	***	0.005	0.002		− 0.469	0.004	***
Income ineq	− 0.121	0.051		0.166	0.079	*	− 0.128	0.051		0.167	0.079	*
Med. house. income	0.233	0	*	− 0.226	0	*	0.211	0		− 0.273	0	**
Res. segregation	− 0.03	0.003		0.219	0.005	**	− 0.032	0.003		0.219	0.005	**
Urbanicity	− 0.02	0.044		− 0.043	0.067		0.001	0.044		− 0.027	0.068	
Southern region	0.163	0.094		− 0.004	0.141		0.132	0.094		− 0.023	0.143	
N	244			228			244			228		
r2	8%			25%			8%			24%		

**Fig. 2:**
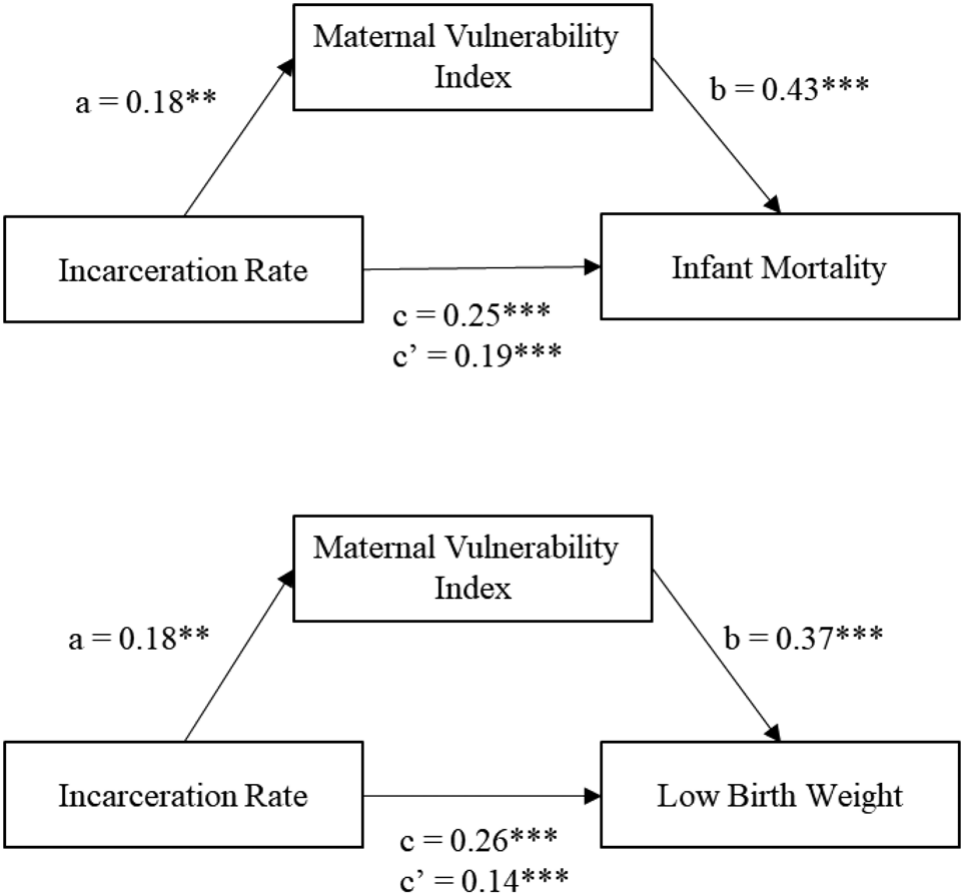
Simple mediation diagram: a, b, c and c’ are path coefficients representing unstandardized regression weights. The c path coefficient represents the total effect of incarceration on low birthweight and infant mortality. The cprime path coefficient refers to the direct effect of incarceration rate on low birthweight and infant mortality. All analyzed paths were significant, **p < 0.05

Table 8 tested the hypothesis of maternal vulnerability mediating the relationship between incarceration and the birth outcomes, which is depicted graphically in Fig. 2. Maternal vulnerability partially mediated the relationship between total incarceration rate and overall low birthweight, as the effect of incarceration on low birthweight decreased when maternal vulnerability was added to the model; however, total incarceration rate (*β* = 0.14, p < 0.001; Table 8) still was a significant predictor of low birthweight. Maternal vulnerability also partially mediated the relationship between total incarceration rate and overall infant mortality. Maternal vulnerability accounted for 41% of the total effect of total incarceration rate on infant mortality, and 47% of the total effect of incarceration rate on low birthweight.

**Table 8:** Mediation results

Type	Effect	Beta	SE	p
Indirect				
	Incarceration rate—MVI—infant mortality	0.186	0	***
	Incarceration rate—MVI—low birthweight	0.138	0	*
Components				
	Incarceration rate—MVI	0.183	0.003	***
	MVI—infant mortality	0.427	0.006	***
	MVI—low birthweight	0.365	0.006	***
Direct				
	Incarceration rate—infant mortality	0.251	0	***
	Incarceration rate—low birthweight	0.194	0	**
Total	Infant mortality	0.329		
	Low birthweight	0.261

## Discussion

The purpose of this study was to examine whether incarceration affects birth outcomes, and birth outcomes separately for infants born to Black women compared to infants born to White women. Findings support the hypothesis that U.S. counties with higher total incarceration rates have a higher prevalence of infant mortality and low birthweight births. These findings suggest that mass incarceration contributes to worse population-level birth outcomes. The hypothesis regarding Black–White disparities was only partially supported as total incarceration rates were associated with a widening of disparities (increasing the Black–White ratio) for low birthweight only. U.S. counties with higher maternal vulnerability scores also have a higher prevalence of infant mortality and low birthweight and decrease Black–White disparities in infant mortality. Finally, the findings partially support the hypothesis that maternal vulnerability mediates the relationship between total incarceration rates and birth outcomes—given the partial mediation.

This study demonstrates that mass incarceration impacts birth outcomes for all birthing people, not just Black women, supporting previous research on the community-level “spillover” effects of mass incarceration. However, total incarceration rates were associated with increased Black–White disparities in low birthweight. While jail and prison incarceration rates have been found in previous studies to affect the health of communities in different ways, in this paper, type of incarceration did not make a substantive difference on birth outcomes. However, the association of prison incarceration rates on birth outcomes was slightly stronger than the association for jail incarceration rates. It is possible that the long sentences associated with prison destabilizes families and communities for greater periods of time, leading to larger gaps in social, emotional, and economic support.

Several U.S. county-level characteristics were included in this study based on their historical significance both to the criminal legal system and racialized geography of the United States (Weidner & Frase, 2003). Although not central to the study hypotheses, we found that southern region and urbanicity, along with residential segregation, median household income, and income inequality were associated with birth outcomes. U.S. counties and states would benefit by prioritizing policy that directs funding to programs to address the social determinants of health, such as social support, health insurance, housing, antipoverty laws, and food security programs, to combat the “double jeopardy” of poor health and high incarceration rates in the U.S. South (Zaller et al., 2017).

There are limitations in this study that should be noted. First, due to the historical legacy of slavery and the complex, punitive, nefarious effects of being Black in the U.S. (Bailey et al., 2017, 2021; Gee & Ford, 2011; Yearby et al., 2022), this study focused on disparities for infants born to Black and White women. Future research should consider the varied sociohistorical contexts of mistreatment and oppression for Latinx, Indigenous, and Asian peoples, and the consequences for contemporary health. Additionally, although the most recent available data was used for the study, not all data were from the same year, with data sources from 2017 to 2021. While we do not expect significant differences because of this limitation due to the nature of population change, it is important to acknowledge. Lastly, our analysis was limited by the number of U.S. counties for which complete data was available. Of the over three thousand U.S. counties in the U.S., our dataset only included data for 266 U.S. counties for infant mortality outcomes and 244 U.S. counties for low birthweight outcomes, as we were limited by U.S. counties that had birth data available. One important policy consideration would be the improvement in pregnancy and birth data availability, especially for infants born to Black women, as data was limited. There may even be an opportunity for public health surveillance systems to collaborate with correctional systems to better document the public health impact of mass incarceration (see Irazola et al., 2024).

Overall, this study found that incarceration contributes to worsening birth outcomes across U.S. counties and that incarceration potentially results in heightened maternal vulnerability as a pathway to worsening infant birth outcomes. These findings demand specific and explicit attention to the fact that the criminal legal system is a public investment that harms public health (Brinkley-Rubinstein & Cloud, 2020; Dumont et al., 2012; National Academies of Sciences, Engineering, and Medicine, 2020) as recently acknowledged by the American Public Health Association (Lead, 2020) and Healthy People 2030 (Social Determinants of Health, Office of Disease Prevention and Health Promotion). One policy-level implication of this study would be a reduction in the overuse of, if not the eradication of, prisons and jails (Lead, 2020). Instead of investing in the criminal legal system, public investment could prioritize increasing social support and social capital within structurally marginalized communities through access to adequate healthcare, safety, education, housing, and employment, to improve health outcomes for all infants.

## Data Availability

All data is publicly available and detailed in methodology.

## Appendix 1: Descriptions of Subtheme Items

- Th1: Reproductive Healthcare: Access to family planning and reproductive care are important determinants of maternal outcomes like mortality. The Reproductive Healthcare theme captures maternal health drivers like access to and funding for reproductive health and family planning (including abortion), availability of skilled attendants (obgyns and certified nurse midwives), and state-level reproductive rights policies.
- Th2: Physical Health: A birthing person’s physical health status entering pregnancy increases their risk of pregnancy complications and/or death. This theme includes indicators of prevalence of non-communicable diseases (hypertension, obesity, and diabetes) and STIs (gonorrhea, syphilis, chlamydia, Hepatitis B, and HIV). It also includes an indicator of self-rated health.
- Th3: Mental Health and Substance Abuse: Pregnancy is a period of high susceptibility when physiological changes can put birthing individuals at increased risk of mental health issues. The mental health and substance abuse theme includes indicators of frequent mental distress, prevalence of mental illness, mental health provider availability, prevalence of smoking, and drug overdose death rate.
- Th4: General Healthcare: The health care system where mothers live contributes to health outcomes before, throughout, and after pregnancy. This theme captures accessibility, affordability, and quality of the healthcare system, as well as preventative careseeking behaviors. Indicators in this theme are insurance coverage, primary care provider utilization, distance to the nearest hospital, the prevention quality overall composite indicator (PQI), and the state’s Medicaid expansion status.
- Th5: Socioeconomic Determinants: Socioeconomic factors are known determinants of health outcomes. This theme captures educational attainment for women of reproductive age, food insecurity and poverty, English proficiency, social support, and a social capital index.
- Th6: Physical Environment: The built environment is a critical factor in understanding population health status generally as well as during the pregnancy journey. This theme includes prevalence of severe housing problems, air pollution, violent crime rate, and two measures of transportation access—access to a private vehicle, and a public transit connectivity index.

Surgo Ventures (2021). The U.S. Maternal Vulnerability Index (MVI).

## Appendix 2

Counties included were located in the following states: Alabama, Arkansas, Delaware, the District of Columbia, Florida, Georgia, Kentucky, Louisiana, Maryland, Mississippi, North Carolina, Oklahoma, South Carolina, Tennessee, Texas, Virginia, and West Virginia.

## Code Availability

We used STATA for all coding purposes.
